# Institutionalization of health impact assessment in China: status quo, challenges and perspectives

**DOI:** 10.3389/fpubh.2025.1691412

**Published:** 2025-11-19

**Authors:** Shenyan Xue

**Affiliations:** Kenneth Wang School of Law, Soochow University, Suzhou, China

**Keywords:** health impact assessment, HIA, institutionalization, China, policy

## Abstract

**Background:**

Health Impact Assessment (HIA) is a critical tool for integrating health into public policy. While China has advanced HIA institutionalization through national legislation, a comprehensive analysis of its implementation across different levels of governance is needed.

**Methods:**

This study conducts a systematic policy analysis of national laws, subnational pilot programs, and technical guidelines to examine the development of HIA in China. The analysis focuses on institutional arrangements, drivers, and barriers identified in key regions, including Zhejiang, Sichuan, and Shanghai.

**Results:**

The analysis reveals that despite innovative subnational models such as Zhejiang’s AI-powered decision-support system, national implementation remains fragmented. Key challenges include unclear institutional mandates, a critical tension between health-led and department-led assessment processes, and limited public participation.

**Conclusion:**

We conclude that sustainable HIA institutionalization in China requires not only dedicated legislation but also deeper integration with the Health in All Policies (HiAP) framework, coupled with robust mechanisms for transparency and accountability. These findings offer a roadmap for China and a comparative case for HIA development in other complex governance systems.

## 1 Introduction

Empirical evidence unequivocally demonstrates that health outcomes are profoundly shaped by social and environmental determinants, many of which are driven by public policy (1). A stark illustration comes from China, where in 2010 alone, transportation-related PM2.5 emissions which linked to lax emission controls were responsible for approximately 116,900 premature deaths (2). Such data underscores the critical need for systematic mechanisms to evaluate the health consequences of policies. HIA has emerged as a key tool for this purpose, designed to identify health risks and benefits and propose mitigating measures (3).

While the technical side of HIA is well-established worldwide, its success in practice depends on institutionalization. This process involves integrating HIA into lasting governance structures, legal frameworks, and standardized procedures. It is at this nexus of technical tool and governance system that a significant knowledge gap exists, particularly within complex, multi-level governance contexts like China. Existing scholarship on China has primarily focused on introducing HIA concepts or its application in specific sectors like urban planning (4). However, there is limited research that analyzes China’s ongoing experiment with HIA institutionalization as a theoretical case in governance (5).

This study addresses this gap by arguing that China’s pathway to HIA institutionalization offers a crucial lens through which to understand broader debates in policy integration and implementation. The Chinese context, characterized by strong central policy directives and adaptive subnational implementation, presents a unique opportunity to examine the tensions between top-down legal mandates and bottom-up institutional innovation. This article conducts a systematic analysis of this process, examining how national strategies and diverse local pilot programs are shaping a distinct model of HIA governance.

By linking the concrete progress of HIA in China to abstract theories of governance, this study aims to make a dual contribution. For a Chinese audience, it provides a synthesized analysis to inform the development of a robust national HIA system (6). For an international audience, it moves beyond a simple case description to offer a comparative framework. China’s progress and challenges in implementing HIA provide important lessons on institutionalizing cross-sectoral policies in complex governance systems, contributing to global scholarship on HiAP and public health governance.

## 2 Status quo of HIA institutionalization in China

In line with legal and policy trajectory regarding to HIA, China has emphasized that safeguarding and improving population health should be a core objective of socioeconomic development, and has advocated for the institutionalization of HIA through legislative measures to ensure its effective implementation (7). Meanwhile, during the pilot phase, various regions across China have actively engaged in localized experimentation, issuing region-specific policies and regulatory documents in an effort to guide the development of HIA system toward institutionalization and effective implementation.

### 2.1 National framework of HIA system

The institutionalization of HIA in China has evolved through a phased, strategy-first approach. In 2016, the *Healthy China 2030 Plan Outline* first called for the comprehensive establishment of an HIA system, marking the formal entry of HIA into national strategic discourse. This was followed by reinforced commitments in key administrative documents, including the *State Council’s Opinion on Deepening the Patriotic Health Campaign* (2020) and *State Council’s Notice on the 14th Five-Year Plan for National Health* (2022), which emphasized technical development and intersectoral coordination (8).

A critical legal milestone was achieved in 2020 with the enactment of the *Basic Law on Medical and Health Promotion of the People’s Republic of China (hereinafter called “Basic Law on health”)*, which explicitly mandates the establishment of HIA and links health outcomes to government performance evaluation (9). Most recently, the *14th Five-Year Plan for National Economic and Social Development and the Outline of the Long-Range Objectives for 2035 (hereinafter called “14th Five-Year Plan and 2035 Vision”)* (2021) introduced a mandatory requirement for public health impact assessment in the context of ecological emergencies, signaling growing recognition of HIA’s role in cross-sectoral risk governance (10). Together, these developments reflect a transition from strategic vision to legal obligation, though implementation mechanisms remain under development.

To map the evolving legal and policy landscape for HIA in China, Table 1 synthesizes key provisions across constitutional, statutory, and strategic instruments. This multi-level analysis reveals a growing normative commitment to institutionalizing HIA, though binding enforcement mechanisms remain limited outside the *Basic Law on health*.^1^

**Table 1:** Legal and policy foundations for HIA in China: a multi-level analysis of constitutional, statutory, and strategic frameworks.

Title of normative instrument	Level & effective year	Key provisions on HIA	Scope of application	Institutional mechanism	Enforcement & accountability	Source of authority & formulation institution
Constitution of the PRC	Constitution (1982, amended)	Art.21: “The State protects the people’s health”; Art.33(3): “The State respects and safeguards human rights”; Art. 45(1): Right to material assistance for health; Art. 26(1): Environmental protection and safeguarding public health	Foundational principle for public health governance	Implies duty of preventive governance	Not directly enforceable, but interpretive basis	Constitutional; National People’s Congress
Basic law on health	National law (2020)	Art. 6: Mandates establishment of HIA system; links health outcomes to government performance evaluation; Art. 71: Requires research on health risk factors and development of preventive measures	Socioeconomic plans, policies, major projects	Government-led, intersectoral coordination	Inclusion in government performance evaluation	Statutory; Standing Committee of the NPC
14th Five-Year Plan and 2035 Vision	National strategy (2021)	Establish a public health impact assessment system for ecological and environmental emergencies	Post-emergency recovery, environmental projects	Coordination between health and environmental agencies	Policy commitment; implementation guidance	Strategic; NPC
Healthy China 2030 Plan Outline	National strategy (2016)	Comprehensively establish an HIA system; Assess potential health impacts of socioeconomic plans, policies, and major projects; Focus on high-risk areas and vulnerable populations	Broad: all major policies and projects; special focus on environmental health risks	Inter-ministerial coordination framework	Monitoring and evaluation framework proposed	Strategic; Central Committee of the Communist Party of China, the State Council
State Council’s Opinion on Deepening the Patriotic Health Campaign	Administrative guidance (2020)	Reaffirms need for HIA in major projects; promotes “health-in-all-policies”	Major infrastructure and public policy	Encourages technical development of HIA tools	Implementation guidance, no binding sanctions	Administrative; the State Council
State Council’s Notice on the 14th Five-Year Plan for National Health	Administrative guidance (2022)	Reiterates HIA development goals; calls for pilot technical schemes for major projects	Major projects, policy formulation	Institutional development and capacity building	Monitoring through health planning systems	Administrative; the State Council

Based on the HIA framework established by the WHO and drawing on relevant experiences from countries with advanced HIA systems, such as the United States, the United Kingdom, Canada, Australia, Finland, and Thailand. This paper constructs an ideal HIA model (11). This model serves as a basis for evaluating the development of China’s HIA system across five key institutionalization dimensions, thereby illustrating its current status (12). Explanations of these evaluation dimensions and the rationale for scoring are provided in Table 2.^2^

**Table 2:** Institutional maturity of HIA in China across five key dimensions.

Dimension	Illustration	Scale rule	Score	Score points
Legal mandate	Is there a national law that explicitly mandates the establishment of an HIA system?	0 = None; 5 = Existence of specific laws or core legal provisions	4	Article 6 of the *Basic Law on Health* explicitly establishes a general mandate, though no dedicated HIA legislation exists.
Scope & coverage	Does the HIA framework cover major policy areas, including planning, policies, and projects? Does it incorporate protections for key populations and regions?	0 = None; 5 = Comprehensive coverage with special attention given to key areas or groups	4	The *14th Five-Year Plan and 2035 Vision,* and Healthy China 2030 outline broad coverage across planning, policies, and projects.
Institutional mechanism	Are there designated leading institutions, cross-departmental coordination mechanisms, and technical standards in place?	0 = None; 5 = Clearly designated leading institutions with collaboration platforms and technical guidelines	3	While directional policies are in place, a unified leading institution and technical standard system remain absent.
Enforcement & accountability	Is HIA integration incorporated into government performance assessments, and are there supervision, review, or accountability mechanisms?	0 = None; 5 = Integration into performance assessments with independent review and accountability mechanisms	3	HIA is referenced in government performance assessments (Article 6), yet specific accountability mechanisms are underdeveloped.
Public participation	Are public consultations, information disclosure, and stakeholder participation required within the HIA process?	0 = None; 5 = Legal requirements for public consultation, information disclosure, and stakeholder participation, implemented through transparent processes	1	Current policies afford minimal attention to public participation, representing a notable institutional shortcoming.

**Figure 1:**
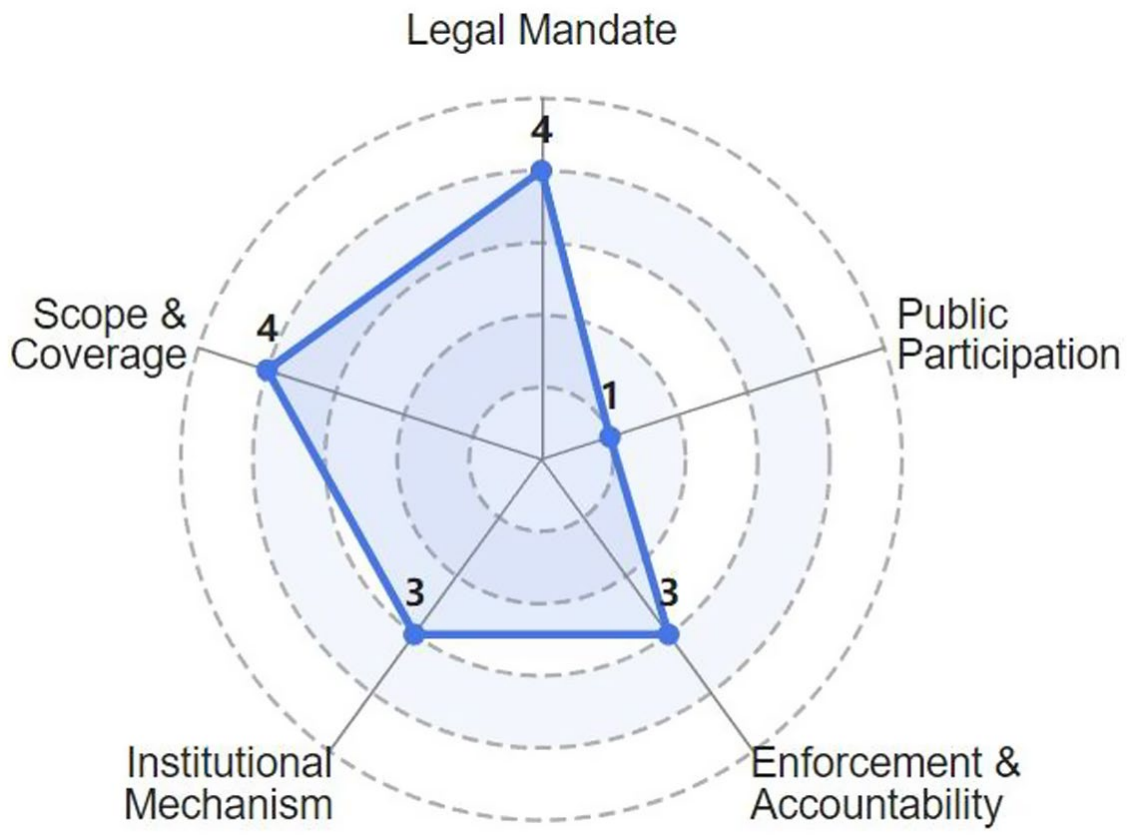
Five-dimensional institutional maturity framework.

To provide a more intuitive assessment of the development level of the HIA system across key institutional dimensions and to identify its strengths and weaknesses, this article develops a HIA system maturity radar chart (Figure 1) based on the preceding analysis.

### 2.2 Pilot attempts towards HIA institution across regions

Beyond national-level institutional design, supported by overarching policy frameworks, various regions in China have actively pursued local legislative initiatives and pilot endeavors to advance the legal institutionalization of HIA (13). Gansu Province, as one of the early adopters of the HiAP approach, proposed the integration of public health considerations into EIA for major and construction projects, and called for the gradual establishment of a health review mechanism, accompanied by pilot initiatives. Subsequently, HIA pilot initiatives have been implemented across various regions (14). By the end of 2024, pilot regions had conducted a total of 1,954 health impact assessments, engaging over 40 government departments, with 76% of the proposed recommendations adopted (15).

Collectively, the regional pilot initiatives mark an evolution from fragmented experimentation toward a more coherent, albeit variegated, system of HIA governance. This transition is characterized by two distinct yet potentially complementary pathways to institutionalization: a techno-managerial model and a strategic-integrative model.

The techno-managerial pathway, exemplified by Zhejiang’s AI-powered decision-support system and the widespread development of standardized assessment tools in Shanghai and Shaanxi, seeks to institutionalize HIA through technical standardization and operational efficiency (16). This approach enhances credibility and scalability by making HIA processes more objective and controllable.

In contrast, the strategic-integrative pathway, demonstrated by Sichuan’s embedding of HIA within its provincial development strategy and Guangxi’s innovations in inter-departmental liaison mechanisms, aims to embed HIA by aligning it with existing political priorities and administrative structures. This approach prioritizes political legitimacy and bureaucratic buy-in over technical precision (17).

The coexistence of these pathways reveals a central tension in HIA institutionalization: between the drive for technical rigor and the imperative of political integration. The most robust implementations, however, suggest a synergy. For instance, Zhejiang’s technological tools provide the data-driven evidence needed to justify the cross-departmental collaboration seen in Guangxi’s model. Thus, the pilot projects not only generate valuable empirical insights but also illustrate the dynamic interplay between different logics of governance that shape HIA’s future in China.

At present, pilot initiatives across various regions have generally reached a consensus that distinct assessment processes should be applied to documents issued by municipal governments and those produced by specific municipal government departments (18). This agreement arises from fundamental differences in institutional design and operational logic between the two types of documents, particularly in three key aspects: (1) the role of the health department in the evaluation process; (2) the allocation of decision-making authority at different stages; and (3) the extent of influence that decisions exert on final outcomes. This paper systematically examines and compares these two assessment models, highlighting the considerable tension within the HIA system between centralized coordination and departmental autonomy (Table 3).

**Table 3:** Comparison of two HIA implementation pathways.

Dimension	Pathway 1: Health Department-led (for municipal government-issued documents)	Pathway 2: Municipal Department-led (for department-issued documents)
Initiating authority	Municipal government, with the health department as lead coordinator	Policy-formulating department initiates independently
Applicable scope	Cross-sectoral or major public policies and plans	Department-specific regulatory or normative documents
Role of health apartment	Lead agency + supervisor + technical advisor (full-cycle involvement in initiation, screening, assessment, and feedback tracking)	Registrar + potential consultant (limited to record-filing; no direct involvement in assessment process)
Decision-making authority by stage		
Whether to conduct HIA?	Health apartment convenes expert panel; standardized methods applied	Department self-assesses (self-screening)
How to conduct HIA?	Implementing entity must report adoption status to health apartment for tracking	Policy-formulating department forms its own panel; methods and quality vary
Adoption of recommendations?	Strong institutionalization, closed-loop management	Formal filing required, but no enforcement or follow-up mechanism
Institutional strength	Strong institutionalization, closed-loop management	Weak institutionalization, relies on departmental initiative
Coverage of HIA	High (systematic screening by professional body)	Low to moderate (risk of selective or omitted assessments)
Quality control	High (standardized procedures, expert oversight)	Variable (depends on departmental capacity and commitment)
Policy influence	High (direct input into early-stage decision-making)	Limited (recommendations often marginalized)

## 3 Challenges HIA institutionalization confronted with in China

Although progress has been made in the institutionalization of HIA in China, there remains a lack of dedicated national legislation or a comprehensive, detailed institutional framework (19). In the absence of explicit legal authorization, local pilot initiatives encounter persistent theoretical debates and practical challenges regarding the legality, feasibility, and effectiveness of HIA implementation.

### 3.1 Fragmentation and institutional gaps in national framework

Although China has affirmed the legal and strategic importance of HIA through a series of national policies and legislative frameworks, its institutional design remains structurally deficient. These shortcomings are most evident in the ambiguity of responsible entities, the absence of standardized procedures, and the lack of meaningful public participation as three core elements essential for effective governance.

While the state mandates that “governments at all levels” establish HIA systems, it fails to designate a lead agency or clearly delineate interdepartmental responsibilities. This institutional ambiguity results in functional overlap and fragmented governance, particularly among key factors such as the health, environmental protection, and development and reform authorities. A salient example is found in major infrastructure projects, where HIA is nominally required to be integrated with EIA. Yet, under the *Environmental Impact Assessment Law*, health risks are routinely treated as peripheral considerations, often reduced to generic statements without systematic or evidence-based analysis.^3^ This reflects not only the marginalization of health in cross-sectoral decision-making but also the absence of an independent HIA initiation mechanism and a dedicated technical authority to ensure methodological rigor (20). Despite possessing the necessary expertise, health departments lack a statutory mandate to participate in early-stage policy formulation. As a result, health considerations are systematically excluded from critical decision points, rendering them institutionally peripheral rather than integral to policy development.

More critically, foundational governance mechanisms have yet to be institutionalized within the HIA framework. These include public participation, information disclosure, and accountability (21). The assessment process remains largely opaque, with no effective avenues for appeal in cases of data falsification or procedural misconduct. This deficit in transparency and oversight undermines the scientific rigor, credibility, and enforceability of the system.

Furthermore, China lacks a unified procedural framework, standardized technical guidelines, or enforceable accountability mechanisms for HIA.^4^ While pilot initiatives in provinces such as Zhejiang, Sichuan, and Guangxi have experimented with diverse implementation models, the resulting variation in assessment processes, methodologies, and organizational structures compromises coherence and limits scalability. In the absence of standardized protocols or national coordination platforms, these efforts remain isolated and noncumulative. Consequently, the significant gap between high-level policy endorsement and weak operational infrastructure confines HIA to symbolic advocacy rather than substantive, institutionalized governance (22).

Equally significant is the underdeveloped legal integration of HIA within broader legal domains, including constitutional, administrative, and criminal law. The absence of a clear legal interface between HIA and these frameworks not only risks inconsistencies in legal interpretation and application but also creates potential regulatory gaps. Such ambiguities may enable institutional rent-seeking and weaken the rule of law, ultimately compromising the legal legitimacy and enforceability of HIA as a public health governance instrument.

### 3.2 Weak authority and narrow coverage in local pilots

Meanwhile, the effectiveness of regional pilot projects on HIA remains limited, constraining their ability to generate practical and replicable experiences that could inform national legislation. This limitation is starkly illustrated by the extremely low coverage of HIA. Although the 32 pilot regions cumulatively conducted 1,954 HIAs by the end of 2024, this number is negligible compared to the volume of public policies enacted. For instance, in Hangzhou city alone, 3,353 public policies were issued in 2024, suggesting that HIA assessments were applied to only a minute fraction of eligible decisions^5^ (15). As observed in other regions, China’s pilot projects generally adopted similar organizational models wherein the health department assumed a leading role.

However, the health department’s capacity to perform this role effectively is severely hampered by a fundamental mismatch between available data and the informational needs of HIA. While China collects vast amounts of public health statistics, the 2023 *National Health Statistics Bulletin* reveals that authoritative, directly relevant data on population health impacts are extremely limited, covering essentially only drinking water quality and ambient air pollution (monitored in 128 cities).^6^ The absence of robust, nationally representative data on the health impacts of other key factors (e.g., nutrition, transportation, housing) forces HIA practitioners to rely on indirect proxies or local studies of varying quality (23). This critical gap in directly applicable health impact data inherently weakens the health department’s technical authority in cross-sectoral deliberations.

Furthermore, the marginalization of health considerations is evident in the domain of construction projects. Statistics reveal a critical disconnect: while EIA for construction projects had an execution rate exceeding 99% between 2000 and 2009 (averaging 263,000 projects annually), the same period saw an average of 600,000 public petitions annually related to environmental issues^7^ (24). This contrast underscores the ineffectiveness of the existing evaluation system in safeguarding health and highlights an urgent, unmet need for HIA.

In practice, even as the statutory authority for HIA, the health department often lacks sufficient institutional power to ensure its recommendations inform core policy decisions. Under current rules, if internal experts judge a policy to pose no significant health risk, the formal HIA process can be waived altogether (25). Moreover, the health department holds no mandate to intervene in assessments or issue binding recommendations. As a result, HIA inputs are frequently overlooked by other departments, reducing the process to a procedural formality. This institutional weakness leaves the health department in a structurally marginalized position, despite its nominal leading role. The resulting operational contradictions undermine the credibility and practical effectiveness of HIA as a governance tool.

A second institutional shortcoming is the framework’s failure to systematically incorporate all public policies with potential health impacts (26). In practice, assessment scope is often artificially narrowed. For example, Zhongshan City’s pilot program defines “public policies” solely as documents issued by the municipal government and non-health departments, thereby excluding health sector policies from evaluation and limiting HIA’s applicability from the outset.

In contrast, Nanning City has attempted to expand coverage through a “minimum assessment indicator system,” which requires each department to submit at least one policy per year for HIA review. While innovative, this approach faces practical challenges: departments retain considerable discretion in selecting which policies to submit, raising questions about representativeness and alignment with HIA objectives. Furthermore, performance pressure may incentivize symbolic compliance. Examples include nominating low-impact policies or conducting superficial assessments, which could further undermine the system’s legitimacy.

## 4 Countermeasures and suggestions

Just as a legal system should constitute an intricate network of interrelated laws, the institutionalization of any system requires logical coherence and functional complementarity among its constituent institutions. To advance the institutionalization of HIA, it is essential to strengthen the overall institutional design, clearly define the system’s scope and boundaries, and establish robust procedural norms. Furthermore, existing legal, administrative, and policy resources should be strategically leveraged to support and accelerate HIA’s integration into the governance framework.

### 4.1 Advancing HIA through HiAP: a well-timed path

The development of a modern HIA system is closely intertwined with the advancement of the HiAP approach, both of which aim to improve population health outcomes. Since the adoption of the *Alma-Ata Declaration* at the International Conference on Primary Health Care in 1978, the HiAP concept has been progressively affirmed and refined through key international milestones. These include the *Ottawa Charter of Health Promotion* (1986), *the Adelaide Statement on Health in All Policies* (2010), the *Rio Political Declaration on Social Determinants of Health* (2011), the *Helsinki Statement on Health in All Policies* from the 8th Global Conference on Health Promotion (2013), and the World Health Organization’s *National Action Framework for Health in All Policies* (27).

In parallel with these global developments, China has actively engaged with and adapted the HiAP framework to its national context. Notably, the *“Healthy China 2030” Initiative*, launched in 2016, explicitly embraces HiAP as a core strategy, emphasizing cross-sectoral collaboration to address social, economic, and environmental determinants of health. This commitment was further reinforced during the *National Health and Wellness Conference* (2016), where HiAP was formally adopted as one of China’s six national health work guidelines. Subsequent policy measures, such as the integration of health impact considerations into urban planning, environmental protection, and poverty alleviation efforts, demonstrate China’s dedication to operationalizing HiAP through concrete mechanisms (28).

Given the growing policy support for HiAP and the instrumental role of HIA in its implementation, the institutionalization of HIA should leverage this critical period of HiAP’s rapid development to advance its own systemic integration and capacity building. By aligning with HiAP’s strategic objectives, HIA can enhance its relevance and effectiveness, ensuring that health considerations are systematically integrated into all relevant policy domains.

### 4.2 Defining HIA’s unique institutional role: beyond EIA

It is universally known that HIA is originated from EIA, within which its initial conceptual framework was developed. But EIA’s narrow focus on toxicological and disease-specific pathways was insufficient to generate comprehensive, cross-sectoral interventions for improving public health, suggests that HIA cannot develop as an independent institutional mechanism unless its unique and irreplaceable value which distincts from EIA and related assessment systems is clearly established (29).

Indeed, the core strength of HIA does not lie merely in its ability to evaluate a broader set of health indicators than EIA, but rather in its reconceptualization of “health,” its distinct evaluative logic, and its transformative role in governance (30). These elements constitute the foundational basis for HIA’s institutional independence. To clarify these differences, the following Table 4 provides a comparative analysis of HIA and EIA.

**Table 4:** A systematic comparison of HIA and EIA.

Dimension	EIA	HIA	Key distinctions
Concept of health	Health as absence of disease or physical harm (biomedical model); Focused on pathogenicity (carcinogenicity, toxicity, etc.)	Health as complete physical, mental, and social well-being (WHO definition); Includes both harm prevention and health promotion (improved well-being, resilience, etc.)	EIA adopts a defensive stance; HIA embraces a proactive and holistic health paradigm
Scope of health impacts	Primarily negative, direct, physiological effects; Limited consideration of indirect or positive impacts	Comprehensive identification of: Positive and negative impacts; Direct, indirect, and cumulative effects; Short-term and long-term/latent effects; Impacts on health equity and vulnerable populations	HIA explicitly addresses distributional justice, identifying how policies may exacerbate or reduce health inequalities
Core analytical elements	Focus on: Pollutant concentration; Exposure levels; Dose–response relationships; Risk probability	Structured assessment of: Nature (positive/negative); Trend; Severity; Magnitude (population size affected); Distribution (geographic and social disparities); Timing (frequency, duration, latency); Likelihood and certainty of evidence	HIA provides a systematic, multi-dimensional framework for describing and evaluating health impacts beyond simple risk estimation, supporting refined decision-making
Domains of health addressed	Mainly: Respiratory diseases; Cancer; Acute poisoning	Broad spectrum including: Physical health; Mental health and well-being; Diet and obesity; Health behaviors; Access to healthcare; Environmental health (green space, noise)	HIA supports HiAP by assessing non-health sector interventions (transport, housing, urban planning, etc.)
Analytical pathways	Linear pathway: Source → Emission → Medium → Exposure → Dose → Effect → Risk	Systemic pathways: Policy/Project → Social determinants (housing, employment, transport) → Health conditions/behaviors → Health outcomes/inequities	HIA captures indirect and structural mechanisms through which policies shape health, going beyond environmental exposure
Public participation	Procedural participation: Public notice; Comment periods; Limited influence on impact identification	Substantive and empowering participation: Community engagement and co-identification of impacts; Representation of marginalized groups; Participatory methods (e.g., focus groups, citizen panels)	HIA treats the public as co-creators of knowledge and policy, enhancing legitimacy and equity
Methodological approach	Predominantly quantitative models: Air/water dispersion modeling; Risk assessment models; Standardized and replicable	Mixed methods: Quantitative (epidemiological data, burden of disease); Qualitative (stakeholder interviews, scenario analysis); Participatory tools (health impact matrices, equity audits)	HIA is context-sensitive, flexible, and socially informed, accommodating uncertainty and value judgments
Institutional purpose and governance value	Regulatory compliance; Environmental risk control; Project-level decision-making	Promoting population health; Reducing health inequities; Enabling cross-sectoral collaboration; Advancing equity-oriented governance	HIA functions not only as a technical tool but as a mechanism for inclusive and socially just policy-making

Table 4 systematically compares HIA and EIA across eight critical dimensions. It reveals that while EIA remains anchored in a narrow, hazard-centered paradigm focused on preventing environmental harm, HIA embodies a broader, equity-driven, and participatory approach to shaping healthier societies. Crucially, HIA extends beyond identifying health risks to actively promoting positive health outcomes and addressing the distribution of impacts across populations. Its methodological richness includes structured assessments of impact nature, scale, distribution, and certainty. Combined with its institutional commitment to public participation, these features underscore its distinct identity as a tool for HiAP governance (31). This systemic divergence implies that simply replicating the EIA legal model would fail to capture HIA’s transformative potential in the Chinese context (12). The capacity of HIA to assess non-environmental determinants of health, to quantify health equity impacts, and to institutionalize meaningful public engagement, represents capabilities essential for addressing China’s evolving public health challenges, such as urbanization-related health disparities and mental health burdens.

### 4.3 Reconfiguring the role of health authorities in HIA: from implementer to systemic enabler

The dual implementation pathways of HIA observed in this study which centralized coordination by health authorities versus decentralized execution by municipal departments reveal a fundamental tension in institutional design: the trade-off between technical legitimacy and governance feasibility. Under the centralized model, although the health department maintains strong procedural control and promotes broader HIA coverage, its dual role as both facilitator and evaluator raises concerns regarding neutrality, particularly in contexts shaped by political pressure or policy alignment incentives. In contrast, the decentralized model, characterized by minimal involvement from the health authority, weakens its ability to safeguard health considerations, often reducing HIA to a voluntary and largely symbolic exercise. This “influence paradox” highlights the limitations of binary governance approaches. These approaches are either comprehensive control or passive oversight, and the paradox points to the need for a more nuanced institutional architecture (32).

To address this challenge, we propose redefining the role of the health authority as a systemic enabler rather than a direct implementer. This involves shifting from “doing HIA” to “ensuring HIA is done well” through three institutional mechanisms. First, the health authority should develop methodological guidance, maintaining a national expert pool and provide training to build capacity across sectors, hereby establishing technical leadership. Second, the health authority should calibrate its degree of engagement in assessments according to the health risk level of policies for proportionate and targeted intervention (33). Third, the health authority should develop an accountability system by linking HIA participation and performance to internal health-sector evaluations and reporting frameworks to enhance the efficacy of HIA.

This repositioning strengthens the health sector’s professional authority while avoiding overreach into domains beyond its mandate. It enables the health authority to act as a knowledge steward and quality assurance body. Future reforms should therefore prioritize legal empowerment, risk-stratified engagement, and transparent knowledge sharing to fully realize the transformative potential of HIA in urban health governance.

### 4.4 Enabling cross-sectoral integration: a proposal for national HIA governance

The institutionalization of HIA in China hinges on the establishment of a robust national coordination mechanism. While local innovations have demonstrated the feasibility of cross-sectoral collaboration, sustainable and equitable implementation across jurisdictions requires centralized leadership and policy coherence. Drawing on successful subnational models such as Zhejiang Province’s Health Zhejiang Construction Leading Group, which operates under joint leadership of the provincial Party Committee and government as a comparable high-level structure should be established at the national level.

Accordingly, the creation of an HIA Coordination Committee under the State Council is strongly recommended. This body would possess the political authority to align health, environmental, economic, and social policies across ministries, ensuring that HIA is systematically integrated into major policy and planning decisions. It would not duplicate the technical functions of the National Health Commission but would instead provide the intersectoral mandate and enforcement power necessary to institutionalize HIA beyond the health sector.

To operationalize this role, the Committee should first issue mandatory, standardized technical guidelines, including screening criteria, equity assessment tools, and health forecasting models to ensure consistency, prevent regulatory arbitrage, and support evidence-based decision-making across regions and sectors. Then, it should establish a centralized digital reporting and monitoring platform, integrated with national e-governance systems, to track assessment completion, recommendation uptake, and inter-agency collaboration in real time. And last, it should embed HIA compliance into inter-ministerial performance evaluations and public accountability mechanisms, thereby institutionalizing cross-sectoral responsibility (34).

Together, this high-level coordination mechanism, unified standards, and transparent monitoring system form an interdependent framework for advancing HIA institutionalization, which balances strategic governance with practical implementation. The health authority’s role as a technical enabler is essential, but only a national coordination body can ensure that HIA becomes a binding, cross-sectoral practice.

### 4.5 Cultivating a culture of HIA: the enabling role of ethical traditions

While institutional change is ultimately driven by political will, legal frameworks, and resource allocation, the cultural environment in which policies take root plays a critical enabling role (35). Values such as equity, public participation, and intergenerational responsibility which are central to HIA do not operate in a cultural vacuum. A supportive normative context can enhance policy legitimacy, shape bureaucratic attitudes, and foster long-term ownership across sectors.

In the Chinese context, certain elements of the Confucian ethical tradition offer a selective resonance with core HIA principles. Confucian concepts like *ren* (benevolence) and *yi* (righteousness) emphasize moral governance and collective well-being. These values align closely with HIA’s preventive and equity-oriented ethos. For instance, Shenzhen’s integration of health compatibility screening into urban planning reflects a practical interpretation of *ren zheng* (benevolent governance), where foresight and care for citizens’ well-being are institutionalized. Similarly, Hangzhou’s interdepartmental coordination mechanisms echo the Confucian ideal of *yi*, which calls for action in the public interest, transcending narrow departmental interests (36).

Yet this resonance is neither automatic nor uncritical. The Confucian emphasis on hierarchy and social harmony may, in practice, constrain the participatory and adversarial dimensions essential to rigorous HIA such as community-led evidence gathering or challenging powerful development agendas. Moreover, the primary drivers of HIA adoption in China today are modern policy imperatives, most notably the Healthy China 2030 strategy rather than a revival of classical philosophy.

Therefore, rather than viewing culture as a foundation for institutionalization, it is more accurate to see it as a strategic resource: one that can be intentionally cultivated to reinforce new institutional norms. By framing HIA not as a foreign import, but as consistent with enduring ethical commitments to public welfare and responsible governance, policymakers can build broader normative support. In this way, cultural narratives do not determine institutional success, but when consciously engaged, they can help embed HIA into the moral imagination of public administration.

Future efforts should therefore go beyond legal and technical reforms to include deliberate cultural work, through leadership discourse, training curricula, and public communication that links HIA to widely shared values. Only then can HIA evolve from a procedural requirement into a lived practice of health-conscious governance.

## 5 Conclusion

### 5.1 Descriptive findings

The institutionalization of HIA in China has progressed through a phased and dual-track approach. At the national level, HIA has gained formal recognition through strategic documents such as the *Healthy China 2030 Plan* Outline and, critically, through legal codification in Article 6 of the *Basic law on health* (2020), which mandates the establishment of an HIA system and links health outcomes to government performance evaluation. Subsequent national strategies, including the 1*4th Five-Year Plan and 2035 Vision*, have further emphasized HIA in contexts such as ecological emergencies. Parallel to these top-down developments, subnational pilot programs particularly in Zhejiang, Sichuan, Shanghai, and Guangxi have advanced localized models of HIA implementation. These include both techno-managerial innovations like AI-powered assessment tools and strategic-integrative approaches as embedding HIA in provincial development plans. Two distinct implementation pathways have emerged: one led by health departments for cross-sectoral policies, and another led by individual departments for internal regulatory documents. Collectively, these efforts reflect a growing, though still fragmented, institutional footprint for HIA across China’s governance system.

### 5.2 Key challenges

Despite this progress, several systemic challenges hinder the effective and equitable implementation of HIA. First, the absence of dedicated national legislation results in ambiguous institutional mandates, particularly regarding leadership, interdepartmental coordination, and enforcement. This ambiguity manifests in a critical tension between health-led and department-led assessment models, leading to inconsistent application and accountability. Second, the lack of standardized technical guidelines, unified procedural frameworks, and robust data infrastructure undermines the scientific rigor and comparability of assessments. Third, public participation remains severely underdeveloped, with no institutionalized mechanisms for stakeholder engagement or information disclosure, limiting democratic accountability. Fourth, the scope of HIA is often arbitrarily narrowed, excluding many policies with significant health implications, while pilot programs cover only a minimal fraction of actual policy outputs. Finally, the health sector, despite its statutory role, lacks sufficient authority and data resources to ensure its recommendations are integrated into decision-making, resulting in HIA being treated as a symbolic rather than substantive process.

### 5.3 Recommendations

To advance sustainable HIA institutionalization, three interdependent reforms are recommended. First, the role of health authorities should be redefined from direct implementer to technical steward: responsible for risk-based screening, methodological oversight, quality assurance, and capacity building, rather than micromanaging every assessment. Second, a high-level HIA Coordination Committee should be established under the State Council to provide cross-sectoral authority, mandate standardized protocols, ensure inter-ministerial compliance, and integrate HIA outcomes into national performance evaluation systems. Third, while institutional and legal reforms are paramount, cultural narratives should be intentionally leveraged to reinforce HIA’s legitimacy. By aligning HIA with widely recognized values such as *ren* (benevolence) and *yi* (righteousness) and embedding these in leadership discourse and civil service training, policymakers can cultivate a long-term culture of health-conscious governance. Ultimately, successful HIA institutionalization in China will depend not only on legal mandates but on the integration of technical credibility, political authority, and normative support within a coherent governance framework.

## Footnotes

^1^All documents were retrieved from official government websites as of 2025. The constitutional provisions are not self-executing but serve as interpretive foundations for statutory and policy development. “Enforcement” refers to mechanisms that ensure compliance, such as performance evaluation, monitoring, or legal sanctions. The *Basic law on health* (2020) is the first national law to mandate HIA, marking a critical step toward legal institutionalization.

^2^Scores (0–5) are based on textual analysis of constitutional, statutory, and policy documents (2016–2022). A score of 5 indicates full development of the institutional element; 0 indicates absence. Data sources: Normative instruments mentioned in Table 1. Public participation remains the weakest dimension, highlighting a critical gap in democratic accountability.

^3^China’s current EIA legal framework provides insufficient attention to health impact assessment. Articles 10 and 17 of the *Environmental Impact Assessment Law* set forth the required content for EIA of plans and construction projects, respectively, but neither explicitly mandates the evaluation of human health effects. This omission is echoed in the technical guidance: the *2016 Technical Guidelines for Environmental Impact Assessment of Construction Projects (General Principles)*, issued by the former Ministry of Environmental Protection, only requires, in clauses 4.2.1, 6.3.6, and 9.4, that projects posing significant potential health risks identify hazard sources, analyze exposure pathways, and establish environmental monitoring plans. These provisions are narrowly scoped to high-risk cases and focus primarily on risk identification and monitoring, without requiring systematic health impact prediction, mitigation measures, or public health risk communication. Consequently, health impact assessment remains outside the standard, comprehensive EIA process.

^4^Even Articles 6 of the *Basic law on health (2020)*, which explicitly calls for the establishment of a HIA system, and the *State Council’s Notice on the 14th Five-Year Plan for National Health (2022)*, which advocates pilot HIA projects, fail to establish concrete procedural mechanisms or binding legal requirements for their implementation.

^5^The national HIA pilot program was launched on July 20, 2021. The number of public policies for Hangzhou in 2024 was obtained from the PKULaw database on September 26,2025 (www.pkulaw.com). The disparity highlights the vast gap between policy output and systematic health assessment.

^6^Data sourced from the *National Health Development Statistical Bulletin* (2023). The key constraint is not a general lack of health data, but the scarcity of official, direct measurements of “health impact” relevant to policy assessment. Monitoring is largely confined to environmental hazards (water, air), leaving other social determinants of health without authoritative, impact-oriented metrics, which compromises the evidence base for HIA.

^7^Data from a doctoral dissertation on HIA in construction project. The high EIA execution rate coupled with a substantial volume of environmental complaints serves as a proxy indicator for the failure of the current system to address health impacts effectively, thereby demonstrating the necessity of HIA.
